# Therapeutic effects of sequential chemoradiotherapy with pemetrexed and cisplatin on locally advanced laryngeal cancer

**DOI:** 10.12669/pjms.325.10640

**Published:** 2016

**Authors:** Youmao Tao, Chong Ma, Xiangdang Yin, Xuedong Fang, Lixiu Liu

**Affiliations:** 1 Youmao Tao, China-Japan Union Hospital, Jilin University, Changchun 130033, China; 2 Chong Ma, China-Japan Union Hospital, Jilin University, Changchun 130033, China; 3 Xiangdang Yin, Jilin Cancer Hospital, Changchun 130001, China; 4 Xuedong Fang, China-Japan Union Hospital, Jilin University, Changchun 130033, China; 5 Lixiu Liu, China-Japan Union Hospital, Jilin University, Changchun 130033, China

**Keywords:** Laryngeal cancer, Pemetrexed, Cisplatin, Chemoradiotherapy

## Abstract

**Objective:**

To explore the therapeutic effects of sequential chemoradiotherapy with pemetrexed and cisplatin on locally advanced laryngeal cancer (LALC).

**Methods:**

Fifty LALC patients who were treated in our hospital between January 2010 and January 2012 were selected and randomly divided into an observation group and a control group (n=25). The two groups were given conventional radiotherapy in the same manner, before which two cycles of chemotherapy were performed. The observation group intravenously infused with 500 mg/m^2^ pemetrexed on d1 and 25 mg/m^2^ cisplatin on d1-3, with 28 days as a cycle. The control group was intravenously infused with 25 mg/m^2^ cisplatin on d1-3 and 400 mg/m^2^ fluorouracil, with 28 days as a cycle. The short-term effects and adverse reactions of both groups were observed after treatment, and their survival was observed by follow-up for five years.

**Results:**

The response rate was 84% (21/25) in the observation group and 64% (16/25) in the control group, between which the difference was statistically significant (P<0.05). The differences in the incidence rates of short-term adverse reactions such as grade III-IV gastrointestinal reactions and bone marrow suppression were not statistically significant between PC regimen (pemetrexed combined with cisplatin) and PF regimen (cisplatin combined with fluorouracil) (P>0.05). The incidence of long-term adverse reactions such as grade III-IV laryngeal edemas, laryngeal cartilage inflammation and laryngeal cartilage necrosis showed no significant differences between the two groups (P>0.05). The median survival was 3.3 years after PC chemotherapy and 2.8 years after PF chemotherapy, between which the difference was not statistically significant (P>0.05). The levels of serum tumor markers significantly decreased after PC and PF treatments compared with those before (P<0.05).

**Conclusion:**

Combining PC chemotherapy with radiotherapy has satisfactory short-term therapeutic effects on LALC, and the resulting adverse effects can be tolerated. Therefore, this strategy is worthy of promotion and application in clinical practice.

## INTRODUCTION

Laryngeal cancer is a common type of malignant tumor. Surgical treatment may be effective for early laryngeal cancer, which, however, is often difficult to achieve the desired results in patients with locally advanced laryngeal carcinoma (LALC). Concurrent chemoradiotherapy is a primary method of treatment for LALC,^1^ with 5-year overall survival rates of 10% to 45%. The standard chemotherapy regimen is cisplatin combined with fluorouracil (PF regimen).^2^ The 50 LALC patients who were treated in our hospital between January 2010 and January 2012 received PC regimen (pemetrexed combined with cisplatin) and PF regimen (cisplatin combined with fluorouracil), and the therapeutic effects and adverse reactions were compared.

## METHODS

### Baseline clinical data

Fifty LALC patients who were treated in our hospital between January 2010 and January 2012 were selected. This study was approved by the ethics committee of our hospital, and written consent was obtained from all patients. They all received full physical examinations, routine blood test, clinical biochemical examination, frontal chest radiography, laryngoscopy and larynx MRI before treatment, with expected survival of over three months. They were all diagnosed as laryngeal squamous cell carcinoma by biopsy and pathological examination. The scores of systemic performance of the patients were 0-2 points. No abnormalities were observed in hepatic and renal functions or electrocardiogram before chemotherapy. There was no serious laryngostasis, and chemotherapy contraindications were excluded. The patients were randomly divided into an observation group and a control group (n=25). The observation group had 20 males and 5 females, aged between 34 and 77 (56.3 ± 2.1) years old. Tumor stages and metastasis: 15 cases of T3, 10 cases of T4, 9 cases of N1, 12 cases of N2 and 4 cases of N3. Tumor locations: 12 cases of glottic cancer, 8 cases of supraglottic cancer and 5 cases of subglottic cancer. There were 19 males and 6 females in the control group, aged between 33 and 76 (54.6 ± 3.2) years old. Tumor stages and metastasis: 14 cases of T3, 11 cases of T4, 8 cases of N1, 12 cases of N2 and 5 cases of N3. Tumor locations: 11 cases of glottic cancer, 9 cases of supraglottic cancer and 5 cases of subglottic cancer. The two groups had similar baseline clinical data (P>0.05).

### Chemotherapy

The observation group received PC chemotherapy regimen: 500 mg/m^2^ pemetrexed was intravenously infused for on d1 for over a half hour and 25 mg/m^2^ cisplatin on d1-3, with 28 days as a cycle; pretreatment should be conducted with dexamethasone before using pemetrexed. The control group was intravenously infused with 25 mg/m^2^ cisplatin on d1-3 and 400 mg/m^2^ fluorouracil on d1-3, with 28 days as a cycle. During chemotherapy, the patients were subjected to fluid infusion as well as diuretic and antiemetic treatments. Radiotherapy was performed after two cycles of chemotherapy.

### Radiotherapy

All patients received radiotherapy using Elekta precise dual-photon linear accelerator, covering primary lesions and the draining areas of lymph nodes. With CT for imitative positioning, the primary lesions and lymph node metastasis were outlined for digital reconstructed radiography (DRR). The multi-leaf collimator technology was used to directly sketch the irradiation range on DRR. A 6 MV photon line was selected, and electron beam irradiation with appropriate energy was applied on the posterior region of neck. Its radiation dose was Dt60-70 Gy/6-7 weeks (2.0 Gy/d, 5 times a week),^3^ and the spinal cord dose was less than 40 Gy. The patients who could receive surgery after radiotherapy for N2 or above lymph node metastasis were subjected to cervical lymph node dissection.

### Observation indices and criteria for therapeutic effects

Laryngeal MRI and electronic laryngoscopy were reexamined one month after treatment to observe the short-term therapeutic effects.^4^ Complete remission (CR): Tumor disappears completely in longer than one month; partial response (PR): the product of the maximum diameter and the maximum perpendicular diameter of the tumor is reduced by over 50% in more than one month; stable: the product is reduced by no less than 50% and increased by no more than 25% in longer than one month; disease progression: the product exceeds 25%, or the patient dies. Response rate (RR) = CR + PR. Survival of the two groups was observed by follow-up for five years, and their adverse reactions were evaluated using the national cancer institute-adverse events commonly using terms.

### Detection of serum tumor markers

Blood samples of the two groups were retained to detect tumor markers using chemiluminescence assay, including cytokeratin 19 fragment (CYFR21-1), carbohydrate antigen 19-9 (CA19-9), carcinoembryonic antigen (CEA) and squamous cell carcinoma-associated antigen (SCCAg).

### Statistical analysis

All data were analyzed by SPSS19.0. The categorical data were expressed as ($$$$ ±s) and compared by t test. The numerical data were expressed as relative numbers and compared with χ^2^ test. P<0.05 was considered statistically significant.

## RESULTS

### Short-term therapeutic effects

RR was 84% (21/25) in the observation group and 64% (16/25) in the control group, between which the difference was statistically significant (χ^2^=5.185, P<0.05) (Table-I).

**Table-I:** Short-term therapeutic effects (case).

*Group*	*CR*	*PR*	*SD*	*PD*
Observation	15	6	3	1
Control	12	4	7	2

### Short-term adverse reactions

For observation and control groups, the incidence of grade III-IV gastrointestinal reactions and bone marrow suppression were 12% (3/25) and 20% (5/25) respectively, those of grade III-IV liver dysfunction were 12% (3/25) and 12% (3/25) respectively, those of grade III-IV kidney dysfunction were 16% (4/25) and 24% (6/25) respectively, and those of grade III-IV laryngeal reactions were 16% (4/25) and 24% (6/25) respectively. The differences in the incidence rates of short-term adverse reactions were not statistically significant between the two groups (χ^2^=1.195, 1.315, 0.592, 1.118, 0.632, P>0.05) (Table-II).

**Table-II:** Short-term adverse reactions (case).

*Group*	*Gastrointestinal reactions*				*Bone marrow suppression*				*Liver dysfunction*				*Kidney dysfunction*				*Laryngeal reactions*			

	*I*	*II*	*III*	*IV*	*I*	*II*	*III*	*IV*	*I*	*II*	*III*	*IV*	*I*	*II*	*III*	*IV*	*I*	*II*	*III*	*IV*
Observation	11	11	2	1	10	12	2	1	9	13	1	2	11	10	2	2	13	8	3	1
Control	12	10	3	0	9	11	3	2	8	14	2	1	10	9	2	4	12	7	4	2
χ^2^	1.194				1.314				0.591				1.117				0.633			
P	>0.05				>0.05				>0.05				>0.05				>0.05			

### Long-term adverse reactions

For observation and control groups, the incidence of grade III-IV laryngeal edemas were 8% (2/25) and 12% (3/25) respectively, those of grade III-IV laryngeal cartilage inflammation were 16% (4/25) and 16% (4/25) respectively, and those of laryngeal cartilage necrosis were 16% (4/25) and 20% (5/25) respectively. The incidence rates of long-term adverse reactions showed no significant differences between the two groups (χ^2^=1.150, 1.281, 0.804, P>0.05) (Table-III).

**Table-III:** Long-term adverse reactions (case).

*Group*	*Laryngeal edema*				*Laryngeal cartilage inflammation*				*L*			

	*I*	*II*	*III*	*IV*	*I*	*II*	*III*	*IV*	*I*	*II*	*III*	*IV*
Observation	13	10	1	1	11	10	3	1	9	12	3	1
Control	10	9	1	2	7	11	2	2	5	12	4	1
χ^2^	1.151				1.284				0.803			
P	>0.05				>0.05				>0.05			

### Survival times

**Fig.1:**
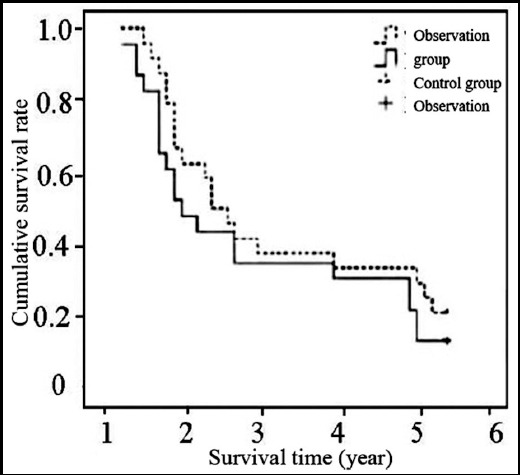
Kaplan-Meler survival curves.

During the 5-year follow-up, four cases in the observation group and three cases in the control group were lost to follow up. Three cases in the observation group and four cases in the control group received cervical lymph node dissection after treatment. After the patients who lost contact were excluded, the 5-year survival rate of the remaining 43 patients was 32.55% (14/43). Such rate of the observation group (33.33% (7/21)) was similar to that of the control group (31.81% (7/22)) (P>0.05). The median survival of the 43 patients was 3.1 years [95% confidential interval (CI): 1.61-3.22]. Such time was 3.3 years [95%CI: 1.92-2.89] after PC chemotherapy and 2.8 years [95%CI: 1.54-3.17] after PF chemotherapy, between which the difference was not statistically significant (P>0.05) (Fig.1).

### Levels of serum tumor markers before and after chemotherapy

The levels of serum tumor markers (CYFR21-1, CA19-9, CEA, SCCAg) significantly decreased after PC and PF treatments compared with those before (P<0.05) (Table-IV).

**Table-IV:** Levels of serum tumor markers before and after chemotherapy.

*Serum tumor marker*	*Observation group (n=25)*		*P*	*Control group (n=25)*		*P*

	*Before treatment*	*After treatment*		*Before treatment*	*After treatment*
CYFR21-1 (ng/ml)	4.77±1.02	2.03±0.55	<0.05	4.87±1.03	2.01±0.53	<0.05
CA19-9 (U/ml)	45.24±7.47	21.13±3.57	<0.05	45.37±7.22	22.04±3.12	<0.05
CEA (ng/ml)	8.77±2.34	3.34±1.01	<0.05	8.97±2.04	3.17±1.04	<0.05
SCCAg (ng/ml)	2.09±1.11	1.14±0.44	<0.05	2.15±0.77	1.13±0.42	<0.05

## DISCUSSION

The incidence of LALC is increasingly high in clinical practice, and the number of LALC patients accounts for about 70% of the total number of laryngeal cancer cases.^5^ Laryngeal cancer is more sensitive to radiotherapy which can maintain the normal structure and vocal function of patients to improve their quality of life.^6^ In case of recurrence after treatment, partial laryngectomy or total laryngectomy can be used, but the methods are not effective for locally advanced laryngeal squamous cell carcinoma.^7^,^8^ Chemotherapy is still one of the main methods, which can elevate the retention rate of the larynx. Previous randomized experimental studies have shown that concurrent chemoradiotherapy was superior to single radiotherapy and postoperative adjuvant radiotherapy, but the side effects should not be neglected.^9^

Cisplatin, as the most commonly used metal platinum complex, is a cell-cycle non-specific anticancer drug. With activities for both aerobic and anaerobic cells, it can enhance the sensitivity to radiotherapy. Cisplatin exhibits antitumor activity mainly through the central axis in the structure, cross-links with DNA or form cross-linking between DNA and protein, so as to inhibit DNA replication, transcription and proliferation of tumor cells.

PF regimen is common in clinical practice for the chemotherapy of head and neck tumors, with the efficiency of about 40%. This regimen enables patients to maintain normal vocal cord and vocal function as well as to improve their quality of life, but fails to prolong the overall survival. Pemetrexed is a plant-derived anticancer drug acting on the M phase, which prevents the recombination of microtubules related to mitosis and other important cellular functions and induces apoptosis by promoting them to aggregate into stable forms and inhibiting their deaggregation. As evidenced by phase II clinical experiments for head and neck squamous cell carcinoma, pemetrexed had high efficiency and tolerance to toxic and side effects. In addition, pemetrexed can evidently augment the sensitivity to radiotherapy, which has been widely used in the treatment of head and neck tumors.^10^-^12^ PC regimen has also become a main way of chemotherapy for head and neck tumors,^13^ but the superiority of its long-term efficacy to that of PF chemotherapy has not been proved hitherto.

Laryngeal cancer may be diagnosed by tumor histocytological or pathological examination. Tumor markers, as an auxiliary examination means of laryngeal cancer diagnosis, play vital roles in laboratory tests and are usually abundant in tumor tissues and serum. Their changes are often of great significance to tumor classification, diagnosis, treatment and prognosis. In the follow-up after treatment, some tumor markers can be used as indices for evaluating the cancer status. During periodic follow-up, tumor recurrence and metastasis can be detected earlier through the detection of tumor markers. CEA is a specific antigen of human embryo, the dynamic changes of which are closely related to tumor therapeutic effects, progression and metastasis. CYFR21-1, CA19-9 and SCCAg are significantly correlated with tumor stage and histological type.^14^-^16^ In this study, after treatment, the CYFR21-1, CA19-9, CEA and SCCAg levels significantly reduced in both groups compared with those before treatment.

The short-term effects of the observation group significantly surpassed those of the control group, with a significantly higher RR. As to the 5-year survival rate, although there was no significant difference between the groups, the median survival of the observation group was longer. Nevertheless, the sample size in this study is small, so the optimum method of administration and dosage of concurrent PC chemotherapy and radiotherapy for LALC patients still need to be further confirmed by multicenter clinical trials with larger sample sizes.

## References

[1] Osborn HA, Hu A, Venkatesan V, Nichols A, Franklin JH, Yoo JH (2011). Comparison of endoscopic laser resection versus radiation therapy for the treatment of early glottic carcinoma. J Otolaryngol Head Neck Surg.

[2] Kerr P, Mark Taylor S, Rigby M, Myers C, Osborn H, Lambert P (2012). Oncologic and voice outcomes after treatment of early glottic cancer:transoral laser microsurgery versus radiotherapy. J Otolaryngol Head Neck Surg.

[3] Tong CC, Au KH, Ngan RK, Cheung FY, Chow SM, Fu YT (2012). Definitive radiotherapy for early stage glottic cancer by 6 MV photons. Head Neck Oncol.

[4] Paluszczak J, Misiak P, Wierzbicka M, Wozniak A, Baer-Dubowska W (2011). Frequent hypermethylation of DAPK, RARbeta, MGMT, RASSF1A and FHIT in laryngeal squamous cell carcinomas and adjacent normal mucosa. Oral Oncol.

[5] Murai R, Yoshida Y, Muraguchi T, Nishimoto E, Morioka Y, Kitayama H (2010). A novel screen using the Reck tumor suppressor gene promoter detects both conventional and metastasis-suppressing anticancer drugs. Oncotarget.

[6] Li D, Feng J, Wu T, Wang Y, Sun Y, Ren J (2013). Long intergenic noncoding RNA HOTAIR is overexpressed and regulates PTEN methylation in laryngeal squamous cell carcinoma. Am J Pathol.

[7] Minor J, Wang X, Zhang F, Song J, Jimeno A, Wang XJ (2012). Methylation of microRNA-9 is a specific and sensitive biomarker for oral and oropharyngeal squamous cell carcinomas. Oral Oncol.

[8] Hakeem AH, Tubachi J, Pradhan SA (2013). Significance of anterior commissure involvement in early glottic squamous cell carcinoma treated with trans-oral CO_2_laser microsurgery. Laryngoscope.

[9] Spielmann PM, Majumdar S, Morton RP (2010). Quality of life and functional outcomes in the management of early glottic carcinoma:a systematic review of studies comparing radiotherapy and transoral laser microsurgery. Clin Otolaryngol.

[10] Wang TS, Li FY, Wang SQ (2011). Treatment of advanced non-small cell lung cancer with docetaxel in combination with tegafur, gimeracil and oteracil porassium capsules. Military Med J South China.

[11] Al-Mamgani A, van Rooij PH, Mehilal R, Verduijn GM, Tans L, Kwa SL (2014). Radiotherapy for T1a glottic cancer:the influence of smoking cessation and fractionation schedule of radiotherapy. Eur Arch Otorhinolaryngol.

[12] Kim TG, Ahn YC, Nam HR, Chung MK, Jeong HS, Son YI (2012). Definitive radiation therapy for early glottic cancer:experience of two fractionation schedules. Clin Exp Otorhinolaryngol.

[13] Wang WX (2012). Thyroid metastasis of occult breast cancer:Case report and literature review. Military Med J South China.

[14] Hanada S, Nishiyama N, Mizuguchi S, Yamano S, Kakehashi A, Wei M (2013). Clinicopathological significance of combined analysis of cytokeratin19 expression and preoperative serum CYFRA21-1 levels in human lung squamous cell carcinoma. Osaka City Med J.

[15] Sheng X, Du X, Zhang X, Li D, Lu C, Li Q (2009). Clinical value of serum HMGB1 levels in early detection of recurrent squamous cell carcinoma of uterine cervix:comparison with serum SCCA, CYFRA21-1, and CEA levels. Croat Med J.

[16] Molina R, Filella X, Auge JM, Fuentes R, Bover I, Rifa J (2003). Tumor markers (CEA, CA 125, CYFRA 21-1, SCC and NSE) in patients with non-small cell lung cancer as an aid in histological diagnosis and prognosis. Comparison with the main clinical and pathological prognostic factors. Tumour Biol.

